# Digital Information Technology Use, Self-Rated Health, and Depression: Population-Based Analysis of a Survey Study on Older Migrants

**DOI:** 10.2196/20988

**Published:** 2021-06-14

**Authors:** Anne Kouvonen, Laura Kemppainen, Eeva-Leena Ketonen, Teemu Kemppainen, Antero Olakivi, Sirpa Wrede

**Affiliations:** 1 Faculty of Social Sciences University of Helsinki Helsinki Finland; 2 Research Institute of Psychology SWPS University of Social Sciences and Humanities Wroclaw Poland; 3 Centre for Public Health Queen's University Belfast Belfast United Kingdom; 4 Department of Geosciences and Geography University of Helsinki Helsinki Finland

**Keywords:** digital information technology, older adults, migrants, health, depression, mobile phone

## Abstract

**Background:**

Previous studies have found that in general, poor health is associated with a lower likelihood of internet use in older adults, but it is not well known how different indicators of health are associated with different types of digital information technology (DIT) use. Moreover, little is known about the relationship between health and the types of DIT use in older ethnic minority and migrant populations.

**Objective:**

The aim of this study is to examine the associations among depressive symptoms and self-rated health (SRH) with different dimensions of DIT use in older migrants.

**Methods:**

We analyzed data from the Care, Health and Ageing of Russian-speaking Minority (CHARM) study, which is based on a nationally representative sample of community-dwelling, Russian-speaking adults aged 50 years or older residing permanently in Finland (men: 616/1082, 56.93%; age: mean 63.2 years, SD 8.4 years; response rate: 1082/3000, 36.07%). Data were collected in 2019 using a postal survey. Health was measured using depressive symptoms (measured using the Center for Epidemiologic Studies Depression Scale) and SRH. Binary logistic regression analyses were used to investigate the associations between the two health indicators and the following six outcomes: daily internet use, smartphone ownership, the use of the internet for messages and calls, social media use, the use of the internet for personal health data, and obtaining health information from the internet. A number of sociodemographic and socioeconomic factors were controlled for in the logistic regression regression analysis. Analyses were performed with weights accounting for the survey design and nonresponse.

**Results:**

After adjusting for sociodemographic and socioeconomic factors, depressive symptoms (odds ratio [OR] 2.68, 95% CI 1.37-5.24; *P*=.004) and poor SRH (OR 7.90, 95% CI 1.88-33.11; *P*=.005) were associated with a higher likelihood of not using the internet daily. Depressive symptoms (OR 1.88, 95% CI 1.06-3.35; *P*=.03) and poor SRH (OR 5.05, 95% CI 1.58-16.19; *P*=.006) also increased the likelihood of smartphone nonuse. Depressive symptoms were additionally associated with a lower likelihood of social media use, and poor SRH was associated with a lower likelihood of using the internet for messaging and calling.

**Conclusions:**

Poor SRH and depressive symptoms are associated with a lower likelihood of DIT use in older adults. Longitudinal studies are required to determine the directions of these relationships.

## Introduction

### Background

The use of digital information technology (DIT) has become an essential and pervasive part of daily living for all generations. However, the so-called digital divide continues to exist because certain segments of the population still cannot access the latest information technologies [1]. In general, there are still large differences between age and cohort groups: although virtually all young and middle-aged adults now use the internet, a considerable proportion of older adults, especially those aged above 75 years, do not have access to the web [2]. The other determinants of DIT use include socioeconomic factors such as education, income, and poverty [3-5], as well as ethnic background [4-8] and migrant status [8]. There is a strong link between traditional social exclusion and digital exclusion [9].

In addition, health is associated with DIT use. This becomes particularly relevant in older age, when health issues can increasingly hamper daily and social activities. A large survey of a representative sample of older adults in 17 European countries showed that better self-rated health (SRH) was associated with an increased likelihood of internet use [3]. This result has been confirmed in other studies [10-19]. Similarly, lower depression levels [5,20-22], better functional capacity [14], better well-being and quality of life [23-25], healthier lifestyles [23,26], fewer chronic medical conditions [5], and more favorable cardiovascular risk factors [27] have been associated with a higher likelihood of DIT use in older adults. In contrast, some studies have found no independent association between health status and internet use [4,13].

However, previous studies included several limitations. First, internet use has typically been assessed as a binary variable, although it comprises vastly different facets and dimensions. Health may affect different aspects of DIT use in different ways. Older adults use the internet in diverse ways, and smartphone use has increased dramatically over the last 5 years; both have implications for the patterns of internet use. Indeed, studies measuring the scope and heterogeneity of DIT use by older adults have been called for [3,17,25,28,29]. Second, many studies have measured health with only one indicator; this can be problematic because studies that have used a variety of physical and mental health and functioning indicators have shown differing associations with DIT use [11].

Third, *older adults* are becoming an increasingly diverse population group. In particular, the number and proportion of older migrants are increasing rapidly in many countries. Despite of this, older migrants have typically been overlooked in studies investigating health and DIT use. Moreover, there are only limited studies on DIT use in ethnic minority older populations other than African Americans and Hispanics in the United States [6,12].

### Objective

To address these limitations, the aim of this study is to examine the associations among depressive symptoms and SRH with different dimensions of DIT use in a representative sample of older Russian-speaking migrants in Finland. Theoretically, this study is placed in the framework of multidimensional and intersectional forms of digital and social exclusion. Intersecting domains such as older age, ill-health, and a migrant background create a higher risk of social exclusion [30,31]. In a rapidly digitizing society, digital exclusion is becoming an increasingly important aspect of social exclusion; in this regard, older migrants with poor health may be a particularly vulnerable group. The so-called double jeopardy hypothesis states that older migrants are in danger of facing exclusion risks because of their age and migrant background [32]. If we add poor health to the mix, older migrants with poor health can be seen as being subject to triple jeopardy. However, there are differences in terms of the type and purpose of internet use. Because older adults engage in a diverse range of internet activities, the different types of uses are likely to be influenced by different (health and other) characteristics [5].

## Methods

### Data

The Care, Health and Ageing of Russian-speaking Minority in Finland (CHARM) study focuses on Russian-speaking community-dwelling older adults (aged 50 years or older) who reside permanently in Finland. People born in Russia or the former Soviet Union constitute the largest migrant group in Finland, accounting for approximately 20% of the total migrant population. The study examines issues related to health and well-being, public service experiences, digital inclusion, and access to different types of care. Data were collected in 2019. A random sample of 3000 people was drawn from the Population Registry, which covers all persons registered as living in Finland. The sample was stratified by gender. Of those invited, a total of 36.07% (1082/3000) of people (men: 616/1082, 56.93%; age: mean 63.2 years, SD 8.4 years) agreed to participate in the study. The participants were asked to answer the questionnaire in Russian or Finnish. A total of 82 participants responded on the web, and the rest responded through a postal survey. The survey responses were weighted to adjust for nonresponse bias. The Finnish Tax Administration register data from 2017 were used to model response propensity. The data included information on earnings and capital income, unemployment benefits, earnings-related and national pensions, and student benefits.

Participation was voluntary, and the participants were informed of their right to withdraw at any time without any consequences. The study protocol was approved by the University of Helsinki Ethical Review Board in the Humanities and Social and Behavioural Sciences (#6/2019). The study conformed to the principles embodied in the Declaration of Helsinki.

### DIT Use

The frequency of internet use was measured on a 7-point scale with response options ranging from “never” to “several times a day.” For the analysis, this variable was dichotomized; the last 2 categories (“once a day” and “several times a day”) were merged to indicate daily internet use (vs others).

A dichotomized (yes or no) follow-up question captured the different types of internet use among the users. The following items were deemed potentially the most relevant for health and were therefore included in this study: messages and calls, social media, accessing personal health data, and obtaining health information. Messages, calls, and social media use relate to social relationships and social connectedness and interaction, which are well-established determinants of health, and accessing personal health data and obtaining health information relate to a person’s health matters. Smartphone use was assessed with a dichotomous question—“Do you own a smartphone?”

### Health Indicators

The CHARM questionnaire included a number of health indicators, such as depressive symptoms; SRH; doctor-diagnosed diseases; limiting long-term illness; physical functioning measured as having difficulties in walking up 3 flights of stairs and difficulties in walking approximately half a kilometer without breaks with or without a walker or walking stick; and reporting hearing, vision or memory, concentration, and learning difficulties. Of these, for this study, we selected the 2 generic indicators that most widely and comprehensively cover physical and mental health—SRH and depressive symptoms.

The 8-item Center for Epidemiologic Studies Depression Scale [33] was used to measure depressive symptoms, with a score of 9 or more points indicating depressive symptoms. The scale and cut-off have been validated for use in community-dwelling older adults [34].

SRH (“In general, would you say your health now is...?”) was assessed on a 5-point scale (1=good; 2=fairly good; 3=average; 4=fairly poor; 5=poor). The groups reporting fairly poor (n=76) or poor (n=28) SRH were merged because of the low numbers of participants in these categories. The single-item SRH measure has been shown to be a valid and reliable instrument that strongly predicts mortality [35,36].

### Covariates

Sex, age, marital status (married or cohabiting vs other), educational qualification in Finland (yes or no), educational level in the country of origin, proficiency in local languages, Finnish citizenship (yes or no), the receipt of income support (yes or no), and the type of survey participation (web-based or postal survey) were included as covariates. The highest educational level in the country of origin was categorized as having no education or basic education, vocational training, or higher education. This categorization is based on the structure of the educational system in the former Soviet Union (the participants were of school age during Soviet times and completed their education mainly in that country because most had moved to Finland as adults).

Acculturation was measured by the degree to which the participant had learned the local official language and whether they had obtained Finnish citizenship. The participants assessed their proficiency in local languages on a 4-point scale. The response options “I use Finnish or Swedish language in various ways in different situations” and “I can participate on everyday conversations in Finnish or Swedish” were categorized as having good proficiency. The response options “I can cope with simple everyday situations in Finnish or Swedish” and “I do not speak either language at all” were merged to indicate poor proficiency. Income support receipt was used to measure poverty. Income support is a means-tested, last-resort financial assistance benefit in Finland [37].

### Statistical Analysis

Descriptive analyses of the sample included bivariate comparisons (frequencies) of sociodemographic characteristics and health indicators (depressive symptoms and SRH) between those who used DIT and those who did not and testing for any differences between these groups (using the chi-square test). Binary logistic regression analysis was used to examine the associations between the health indicators and DIT use. We estimated odds ratios (ORs) and their 95% CIs for different types of DIT use by the health indicators by first controlling for age and sex and then further controlling for marital status, educational attainment, proficiency in local languages, Finnish citizenship, income support, and the type of participation (web-based or postal survey). Sensitivity analyses were performed on the unweighted sample. The two health indicators were not entered simultaneously into the analyses. Health indicators may partly overlap, and entering them simultaneously into the models can cause overadjustment.

The analyses were conducted using Stata, version 15.1 (StataCorp LLC).

## Results

### Descriptive Statistics

Table 1 shows the characteristics of the sample and the distribution of the study variables by daily internet nonuse and smartphone nonuse. Most of the participants (796/1067, 74.6%) were married or cohabiting, 39.97% (400/1082) had obtained some educational qualifications in Finland, and 50% (541/1082) had acquired a higher education in their country of origin. Half of the participants had Finnish citizenship, and 37.78% (385/1019) rated their local language (Finnish or Swedish) skills as good. Deprivation levels were very high because 41.56% (421/1013) of the participants had received income support. Of the participants, 20.08% (194/966) reported depressive symptoms. Half of the participants had average SRH, 26.24% (281/1071) saw their health as fairly good, and 14.01% (150/1071) as good, whereas 9.71% (104/1071) reported that they had fairly poor or poor SRH.

**Table 1:** Characteristics of the study sample and the prevalence of digital information technology nonuse.

Characteristics			Total, n^a^ (%)		Internet nonuse				*P* value^b^			Smartphone nonuse						*P* value^b^	
					Value, N		Value, n (%)					Value, N			Value, n (%)				
**Sex**										.36									.32
	Female	466 (43.07)		458		61 (13.32)					404			68 (16.83)					
	Male	616 (56.93)		609		76 (12.48)					561			81 (14.44)					
**Age (years)**										<.001									<.001
	50-64	653 (60.35)		646		40 (6.19)					614			51 (8.31)					
	≥65	429 (39.65)		421		97 (23.04)					351			98 (27.92)					
**Married or cohabiting**										.006									.18
	No	271 (25.40)		265		46 (17.36)					229			44 (19.21)					
	Yes	796 (74.60)		789		85 (10.77)					726			102 (14.05)					
**Education in Finland**										<.001									<.001
	No	682 (63.03)		670		110 (16.42)					597			118 (19.77)					
	Yes	400 (39.97)		397		27 (6.80)					368			31 (8.42)					
**Education in the country of origin**										<.001									.34
	General, none, or missing	82 (7.58)		79		22 (27.85)					60			11 (18.33)					
	Vocational	459 (42.42)		452		73 (16.15)					405			71 (17.53)					
	Higher	541 (50)		536		42 (7.84)					500			67 (13.40)					
**Proficiency in local languages**										<.001									<.001
	Poor	634 (62.22)		623		102 (16.37)					562			110 (19.57)					
	Good	385 (37.78)		382		23 (6.02)					353			30 (8.50)					
**Finnish citizenship**										<.001									.001
	No	546 (50.98)		538		94 (17.47)					478			96 (20.08)					
	Yes	525 (49.02)		519		39 (7.51)					479			49 (10.23)					
**Income support**										<.001									<.001
	Yes	421 (41.56)		415		71 (17.11)					362			77 (21.27)					
	No	592 (58.44)		583		43 (7.38)					554			61 (11.01)					
**Type of participation**										<.001									.05
	Postal survey	1000 (92.42)		985		136 (13.81)					884			144 (16.29)					
	Web-based survey	82 (7.58)		82		1 (1.22)					81			5 (6.17)					
**Depression**										.003									.02
	Yes	194 (20.08)		189		36 (19.05)					169			39 (23.08)					
	No	772 (79.92)		764		67 (8.77)					717			93 (12.97)					
**Self-rated health**										<.001									<.001
	Fairly poor or poor	104 (9.71)		102		24 (23.52)					87			31 (35.63)					
	Average	536 (50.05)		529		80 (15.12)					463			76 (16.41)					
	Fairly good	281 (26.24)		277		23 (8.30)					265			32 (12.08)					
	Good	150 (14.01)		150		7 (4.67)					141			7 (4.96)					

^a^N varies by variable depending on the number of missing values.

^b^Differences in the prevalence of digital information technology nonuse between groups (from two-tailed chi-square test). *P* values are weighted.

Of the 1082 participants, only 137 (12.66%) did not use the internet daily. Daily internet nonuse and smartphone nonuse did not differ between men and women. Both internet nonuse and smartphone nonuse were much more prevalent among individuals aged 65 years or older (internet nonuse: 97/421, 23%; smartphone nonuse: 98/351, 27.9%) than in the younger age group (internet nonuse: 40/646, 6.2%; smartphone nonuse: 51/614, 8.3%). However, it is notable that for the smartphone use question, the number of missing values was very high, particularly in the older age group (78/429, 18.2%). This may be due to the formulation of the question, which required the participant to circle “Yes” if they owned a smartphone and “No” if they did not. The same question included 4 other items: a tablet, other computer, safety bracelet or safety phone, and web-based banking ID. Some of the participants had only ticked “Yes” to mark the items that they possessed but failed to tick “No” for those that they did not possess. We conducted a sensitivity analysis by recoding these cases as “No” for smartphone use, which increased the prevalence of nonuse to 13.8% (90/653) in working-age older adults and to 41% (176/429) in those aged 65 years or older. It is likely that the actual prevalence of smartphone nonuse is therefore somewhere between 7.8% (51/653) and 13.8% (90/653) in those aged 50-64 years and between 22.8% (98/429) and 41% (176/429) in those aged 65 years or older.

Participants who were married or cohabiting were more likely to use the internet daily, but there was no difference between these groups in terms of smartphone use. Similarly, those who had obtained qualifications in Finland, those who had obtained higher education in their country of origin, those with good local language skills, and those with Finnish citizenship were more likely to use the internet and own a smartphone. In addition, poverty, measured as income support receipt, increased the prevalence of DIT nonuse.

Both daily internet nonuse and smartphone nonuse were more common among the participants who reported depressive symptoms: out of the 189 participants, 36 (20.1%) with depressive symptoms did not use the internet daily compared with 8.8% (67/764) of the participants with no depressive symptoms. The corresponding figures for smartphone ownership were 23.1% (39/169) and 12.9% (93/717), respectively. A similar picture emerged for SRH: compared with those in good health, those with less than good health were more often nonusers of DIT, and the poorer the health, the more common the nonuse.

Of the different types of internet use, the most common was using the internet for calls and messages (n=855). In addition, using the internet to search for health information (n=650) and social media use (n=631) were common. Compared with these, using the internet to view personal health data was much less commonly reported (n=353).

### Health and DIT Use

Table 2 shows the results of the logistic regression models examining the associations between health indicators and daily internet nonuse, and Table 3 presents the associations between health indicators and smartphone nonuse. As shown in Tables 2 and 3, after full adjustments, depressive symptoms were associated with both daily internet nonuse (OR 2.68, 95% CI 1.37-5.24; *P*=.004; Table 2) and smartphone nonuse (OR 1.88, 95% CI 1.06-3.35; *P*=.03; Table 3). Similarly, poor SRH was associated with daily internet nonuse (OR 7.90, 95% CI 1.88-33.11; *P*=.005; Table 2) and smartphone nonuse (OR 5.05, 95% CI 1.58-16.19; *P*=.006; Table 3). Compared with those with good SRH, those with fairly good or average SRH also reported more daily internet nonuse, but there were no associations between these categories and smartphone nonuse.

**Table 2:** Associations between health indicators and daily internet nonuse.

Health indicator			Model 1^a^				Model 2^b^		
			OR^c^ (95% CI)		*P* value		OR (95% CI)		*P* value
**Depression**									
	No	1.00 (reference)		N/A^d^		1.00 (reference)		N/A	
	Yes	2.37 (1.28-4.38)		.006		2.68 (1.37-5.24)		.004	
**Self-rated health**									
	Good	1.00 (reference)		N/A		1.00 (reference)		N/A	
	Fairly good	6.05 (1.62-22.55)		.007		5.44 (1.50-19.81)		.01	
	Average	6.62 (1.86-23.60)		.004		4.77 (1.32-17.26)		.02	
	Fairly poor or poor	9.66 (2.40-38.81)		.001		7.90 (1.88-33.11)		.005	

^a^Adjusted for sex and age.

^b^Additionally adjusted for marital status, education in Finland, education in country of origin, proficiency in local languages, citizenship, income support, and type of participation.

^c^OR: odds ratio.

^d^N/A: not applicable.

**Table 3:** Associations between health indicators and smartphone nonuse.

Health indicator		Model 1^a^		Model 2^b^	
		OR^c^ (95% CI)	*P* value	OR (95% CI)	*P* value
**Depression**					
	No	1.00 (reference	N/A^d^	1.00 (reference)	N/A
	Yes	1.79 (1.02-3.15)	.04	1.88 (1.06-3.35)	.03
**Self-rated health**					
	Good	1.00 (reference)	N/A	1.00 (reference)	N/A
	Fairly good	1.38 (0.49-3.9	.55	1.28 (0.44-3.70)	.65
	Average	2.12 (0.78-5.71)	.14	1.74 (0.62-4.85)	.29
	Fairly poor or poor	6.06 (2.00-18.41)	.002	5.05 (1.58-16.19)	.006

^a^Adjusted for sex and age.

^b^Additionally adjusted for marital status, education in Finland, education in country of origin, proficiency in local languages, citizenship, income support, and type of participation.

^c^OR: odds ratio.

^d^N/A: not applicable.

Table 4 and Table 5 display the associations between health indicators and types of internet use among those participants who reported using the internet. Depressive symptoms were associated with a higher likelihood of social media nonuse (OR 1.93, 95% CI 1.24-2.98; *P*=.003), and poor SRH was associated with a lower likelihood of using the internet for calls and messages (OR 5.27, 95% CI 1.50-18.55; *P*=.01). In terms of the other types of internet use, there were no differences by depressive symptoms or SRH.

**Table 4:** Associations between health indicators and types of internet nonuse: messages and calls and social media among all internet users.

Health indicator		Messages and calls nonuse					Social media nonuse					
		Cases		OR^a,b^ (95% CI)	*P* value	Cases				OR^b^ (95% CI)		*P* value
		Value, N	Value, n (%)			Value, N		Value, n (%)				
**Depression**												
	No	652	68 (10.43)	1.00 (reference)	N/A^c^	670		224 (33.43)	1.00 (reference)		N/A	
	Yes	169	32 (18.94)	1.43 (0.78-2.61)	.25	167		75 (44.91)	1.93 (1.24-2.98)		.003	
**Self-rated health**												
	Good	124	6 (4.84)	1.00 (reference)	N/A	124		31 (25)	1.00 (reference)		N/A	
	Fairly good	239	24 (10.04)	2.28 (0.72-7.25)	.16	244		92 (37.70)	1.30 (0.71-2.37)		.40	
	Average	424	54 (12.74)	1.64 (0.56-4.85)	.37	433		157 (36.26)	1.24 (0.69-2.22)		.47	
	Fairly poor or poor	80	19 (23.75)	5.27 (1.50-18.55)	.01	79		34 (43.04)	1.67 (0.81-3.43)		.17	

^a^OR: odds ratio.

^b^Adjusted for sex, age, marital status, education in Finland, education in the home country, proficiency in local languages, citizenship, income support, and type of participation.

^c^N/A: not applicable.

**Table 5:** Associations between health indicators and types of internet nonuse: personal health data and health information among all internet users.

Health indicator		Personal health data nonuse								Health information nonuse					
		Cases			OR^a,b^ (95% CI)		*P* value		Cases				OR^b^ (95% CI)		*P* value
		Value, N	Value, n (%)					Value, N			Value, n (%)				
**Depression**															
	No	646	410 (63.47)	1.00 (reference)		N/A^c^		663			228 (34.39)	1.00 (reference)		N/A	
	Yes	162	94 (58.02)	0.83 (0.54-1.27)		.39		166			53 (31.93)	1.09 (0.69-1.72)		.72	
**Self-rated health**															
	Good	120	74 (61.67)	1.00 (reference)		N/A		120			41 (34.17)	1.00 (reference)		N/A	
	Fairly good	234	149 (63.68)	0.96 (0.55-1.65)		.87		242			74 (30.58)	0.64 (0.36-1.14)		.13	
	Average	415	257 (61.93)	0.77 (0.46-1.29)		.32		427			146 (34.19)	0.68 (0.39-1.18)		.17	
	Fairly poor or poor	75	47 (62.67)	0.83 (0.40-1.71)		.61		78			26 (33.33)	0.71 (0.33-1.53)		.38	

^a^OR: odds ratio.

^b^Adjusted for sex, age, marital status, education in Finland, education in the home country, proficiency in local languages, citizenship, income support, and type of participation.

^c^N/A: not applicable.

The results for all covariates in the full models are shown in Multimedia Appendices 1 and 2. The results from the sensitivity analyses using the unweighted sample (Multimedia Appendix 3) were mainly in the same direction as the results obtained with the weighted sample. However, in the unweighted sample, depressive symptoms were associated with a lower likelihood of using the internet for messages and calls, and poor SRH was associated with a lower likelihood of social media use. In addition, average SRH was associated with a higher likelihood of using the internet for personal health data.

## Discussion

### Principal Findings

The aim of this study is to examine the associations between mental and physical health and different facets of DIT use among older Russian-origin migrants in Finland and to determine whether these associations are independent of sociodemographic and socioeconomic factors. Our study confirms that DIT use is now strongly embedded in the everyday lives of older adults: the prevalence of internet use was very high in our sample, with 93.8% (606/646) of adults aged below 65 years and 76.9% (324/421) of those aged 65 years or older using the internet daily. In 2019, Finland scored the highest among all European Union countries on the Digital Economy and Society Index, which is a composite index that summarizes relevant indicators of digital performance and includes the components of connectivity, human capital, use of internet services, integration of digital technology, and digital public services [38].

In our study, depressive symptoms were associated with a higher likelihood of daily internet and smartphone nonuse. These results are in line with earlier studies that have shown an association between internet use and depression in older adults [5,20-22]. A large prospective study among US older adults showed that one pathway explaining this association is that internet use influences depression by decreasing loneliness and social isolation [20]. It is well established that loneliness is associated with depression [39], and DIT use has been shown to protect older adults in poor health from social exclusion [40]. A previous study showed that the relationship between internet use and a higher quality of life was mediated by reduced loneliness [24].

In this study, depressive symptoms were additionally associated with a higher likelihood of social media nonuse. A previous study found that social media can become an increasingly important source of feelings of connectedness in older age when retirement and declining health decrease other forms of social engagement [16]. DIT use can considerably increase older migrants’ ability to maintain and expand their dispersed support networks [41]. However, mental health problems such as depression may also result in decreased initiative to engage in new activities [42] such as social media use.

Similar to previous studies [3,10-19], we found an association between SRH and internet use. In addition, poor SRH was associated with smartphone nonuse. Physical changes such as reduced visual acuity, declining motor skills, and age-related changes in cognitive abilities are likely to affect the ability to use DIT [43], and these changes may be particularly critical in terms of hindering smartphone use.

DIT provides many specific health-related resources for those with health problems. It enables them to search for health information, communicate with their health care providers, access their laboratory results, renew their prescriptions, and seek peer support. Therefore, it could be expected that those with poorer health would be more likely to use the internet to seek health information and access personal health data. Indeed, an earlier study showed that having more chronic conditions increased the odds of internet use for health-related tasks [5]. However, in our study, depression and poor SRH did not have an effect on the search for health information among those who used the internet. This finding is consistent with that of a previous Australian study [15]. Moreover, we did not find an association between health status and the use of the internet to access personal health data.

Overall, our findings suggest that in older migrants, the digital divide still exists not only between adults aged 50-64 years and those aged 65 years or older but also between those with poor mental and physical health and those with good health. It is notable that this divide was evident even in our sample, which consisted of cognitively relatively well-functioning older adults who were able to respond to a lengthy questionnaire (20 pages). In fact, it has been noted that as more and more people access the web, the social gap between the majority (the digitally included) and the minority (those who are still digitally excluded) widens [14]. It seems that those who are digitally excluded therefore become even more socially excluded than before. Indeed, a study conducted in the United Kingdom and Sweden showed that now, when access to the internet is widespread and the proportion of those without internet access has become smaller, nonuse has become more concentrated in the most vulnerable groups. In other words, nonusers in both countries increasingly consist of the most vulnerable segments of the population, that is, older participants, those with the lowest amount of education, those with the poorest health, and those who are most socially isolated [9]. Helsper and Reidolf [9] therefore argue that we now see the emergence of a digital underclass. Older migrants may be particularly vulnerable in this regard because older age, poor health, low socioeconomic status, and a migrant background as intersecting domains increase the risk of social exclusion [30,31].

In our study, we were not able to investigate the mechanisms underlying the associations between health and DIT use. However, based on other studies, it can be assumed that functional limitations that can accompany health problems may pose barriers to DIT use [5,14]. It seems that decreased social isolation and loneliness may be one pathway to alleviating depression [20].

### Strengths and Limitations

Our study included several strengths. First, we considered different dimensions of health. Second, we examined DIT use in a diverse way, including daily internet use, smartphone use, and different types of internet use as outcomes. Third, we were able to adjust for a number of relevant sociodemographic and socioeconomic factors that can affect both health status and DIT use.

However, this study also included some limitations. First, our data were cross-sectional; therefore, the associations found cannot be interpreted as causal relationships. It is likely that the relationship between health and DIT use is bidirectional, additive, and synergistic [14]: good health can increase DIT use, and DIT can improve health and well-being. In a large prospective study using a variety of methods, including matching, internet use was shown to reduce depression, and the effect was the largest for older people who lived alone [20].

Second, all our DIT use and health measures were self-reported, which can cause recall bias and misclassification. Third, our results cannot be generalized to the general population or to other migrant populations. In an earlier study on working-age migrants in Finland, Russian-origin migrants were more transnationally oriented than the other 2 migrant groups—Somalis and Kurds—which is likely to manifest as a higher level of DIT use [44]. Notwithstanding these limitations, this study is an important contribution to the growing literature on health and DIT use in older adults. This study provides results that will illuminate the design of future research to incorporate technology use for a better understanding of the mental and physical health situation of vulnerable populations.

### Conclusions

In Finland, as in other Western countries, DIT use is nearing saturation among older age groups, but digital divides still exist. In this study on older migrants, we found that poor SRH and depressive symptoms were associated with a lower likelihood of DIT use. Older adults with poorer health and limited mobility are often the most socially isolated, and they would particularly benefit from the diverse use of DIT [5].

Medeiros et al [14] have proposed that because DIT use such as exchange of web-based messages demands adequate social and executive functioning, it could be used as a marker of older adults’ functional capacity and depression. Therefore, the monitoring of functional capacity and depression could be done routinely by health professionals during health checkups by simply asking about the patients’ DIT use. However, more importantly, there is an opportunity for the use of big data because this monitoring could be done with artificial intelligence using pattern recognition, and the results could be used in health policy planning [14]. The present results also open up possible health interventions that are now particularly relevant considering the COVID-19 pandemic circumstances.

Longitudinal studies are needed to elucidate the mechanisms underlying the associations between health and DIT use. These types of studies can better inform digital inclusion policies for migrants and older adults. Evidence-based digital inclusion strategies and policies are urgently needed to prevent increasing social exclusion of the digitally excluded populations who are also in other ways the most vulnerable, given that public services are rapidly becoming mainly or solely digitally accessible.

## Appendix

Multimedia Appendix 1 Full models with depression as the main predictor.

Multimedia Appendix 2 Full models with self-rated health as the main predictor.

Multimedia Appendix 3 Coefficients for depression and self-rated health and fully adjusted models without weights.

## Abbreviations

CHARM: Care, Health and Ageing of Russian-speaking Minority in Finland
DIT: digital information technology
OR: odds ratio
SRH: self-rated health
