# Supplementary material for: Digital Information Technology Use, Self-Rated Health, and Depression: Population-Based Analysis of a Survey Study on Older Migrants
Source: J Med Internet Res. 2021 Jun 14;23(6):e20988. doi: 10.2196/20988 (PMC8240805; doi:10.2196/20988)
Supplement: Multimedia Appendix 1 [file jmir_v23i6e20988_app1.docx]

| **Appendix 1. Full models with depression as the main predictor.** | | | | | |  |  |  |  |  |  |  |
| --- | --- | --- | --- | --- | --- | --- | --- | --- | --- | --- | --- | --- |
|  |  |  |  |  |  |  |  |  |  |  |  |  |
|  | **Daily internet non-use** | | **Smartphone non-use** | | **Messages and calls non-use** | | **Social media non-use** | | **Personal health data non-use** | | **Health info non-use** | |
|  | OR^a^ | *P* | OR | *P* | OR | *P* | OR | *P* | OR | *P* | OR | *P* |
| **Depression** | 2.68 | *.004* | 1.88 | *.031* | 1.43 | *.245* | 1.93 | *.003* | 0.83 | *.390* | 1.09 | *.720* |
| **Female** | 1.27 | *.434* | 1.34 | *.236* | 0.97 | *.921* | 0.54 | *.001* | 0.73 | *.070* | 0.71 | *.052* |
| **Age (years)** | 1.12 | *<.001* | 1.11 | *<.001* | 1.12 | *<.001* | 1.09 | *<.001* | 1.04 | *.002* | 1.03 | *.005* |
| **Married or cohabiting** | 0.72 | *.340* | 1.19 | *.538* | 0.66 | *.186* | 1.00 | *.981* | 0.95 | *.802* | 1.00 | *.986* |
| **Education in the country of origin (ref: higher)** | | |  |  |  |  |  |  |  |  |  |  |
| *Vocational education* | 2.79 | *.003* | 1.48 | *.147* | 1.79 | *.055* | 1.15 | *.465* | 1.13 | *.523* | 1.37 | *.100* |
| *General/No education/Missing* | 9.11 | *<.001* | 1.40 | *.526* | 10.76 | *<.001* | 2.39 | *.024* | 1.39 | *.440* | 1.10 | *.812* |
| **Education in Finland** | 0.81 | *.647* | 0.62 | *.170* | 0.79 | *.501* | 0.70 | *.114* | 0.57 | *.003* | 0.59 | *.012* |
| **Good local language proficiency** | 0.60 | *.291* | 0.69 | *.333* | 0.87 | *.756* | 1.45 | *.100* | 0.71 | *.091* | 1.50 | *.076* |
| **Finnish citizenship** | 0.62 | *.251* | 0.95 | *.885* | 0.50 | *.072* | 0.64 | *.046* | 0.57 | *.006* | 0.43 | *<.001* |
| **Income support** | 0.77 | *.403* | 1.44 | *.177* | 1.29 | *.384* | 0.68 | *.046* | 1.14 | *.505* | 0.96 | *.853* |
| **Online respondent** | 0.09 | *.031* | 0.64 | *.390* | 0.42 | *.337* | 0.58 | *.066* | 0.43 | *.007* | 0.50 | *.042* |
| **Intercept** | 0.00 | *<.001* | 0.00 | *<.001* | 0.00 | *<.001* | 0.00 | *<.001* | 0.34 | *.194* | 0.11 | *.007* |

^a^OR, odds ratio.
