# Supplementary material for: Digital Information Technology Use, Self-Rated Health, and Depression: Population-Based Analysis of a Survey Study on Older Migrants
Source: J Med Internet Res. 2021 Jun 14;23(6):e20988. doi: 10.2196/20988 (PMC8240805; doi:10.2196/20988)
Supplement: Multimedia Appendix 2 [file jmir_v23i6e20988_app2.docx]

| **Appendix 2. Full models with self-rated health as the main predictor.** | | | | | |  |  |  |  |  |  |  |
| --- | --- | --- | --- | --- | --- | --- | --- | --- | --- | --- | --- | --- |
|  |  |  |  |  |  |  |  |  |  |  |  |  |
|  | **Daily internet non-use** | | **Smartphone non-use** | | **Messages and calls non-use** | | **Social media non-use** | | **Personal health data non-use** | | **Health info non-use** | |
|  | OR^a^ | *P* | OR | *P* | OR | *P* | OR | *P* | OR | *P* | OR | *P* |
| **Self-rated health (ref: good)** |  |  |  |  |  |  |  |  |  |  |  |  |
| *Fairly good* | 5.44 | *.010* | 1.28 | *.651* | 2.28 | *.161* | 1.30 | *.400* | 0.96 | *.874* | 0.64 | *.132* |
| *Average* | 4.77 | *.017* | 1.74 | *.289* | 1.64 | *.369* | 1.24 | *.471* | 0.77 | *.321* | 0.68 | *.170* |
| *Fairly poor or poor* | 7.90 | *.005* | 5.05 | *.006* | 5.27 | *.010* | 1.67 | *.165* | 0.83 | *.609* | 0.71 | *.380* |
| **Female** | 1.37 | *.244* | 1.40 | *.157* | 0.94 | *.818* | 0.61 | *.005* | 0.73 | *.066* | 0.70 | *.043* |
| **Age (years)** | 1.11 | *<.001* | 1.10 | *<.001* | 1.11 | *<.001* | 1.09 | *<.001* | 1.04 | *.002* | 1.03 | *.006* |
| **Married or cohabiting** | 0.69 | *.230* | 1.26 | *.407* | 0.60 | *.098* | 0.99 | *.944* | 0.94 | *.760* | 0.95 | *.789* |
| **Education in the country of origin (ref: higher)** | | |  |  |  |  |  |  |  |  |  |  |
| *Vocational education* | 2.09 | *.020* | 1.21 | *.475* | 1.59 | *.119* | 1.04 | *.815* | 1.17 | *.408* | 1.37 | *.096* |
| *General/No education/Missing* | 6.09 | *<.001* | 1.19 | *.729* | 9.07 | *<.001* | 2.18 | *.029* | 1.59 | *.275* | 1.04 | *.910* |
| **Education in Finland** | 1.12 | *.783* | 0.66 | *.218* | 0.89 | *.714* | 0.73 | *.136* | 0.56 | *.002* | 0.66 | *.041* |
| **Good local language proficiency** | 0.64 | *.284* | 0.74 | *.379* | 0.87 | *.715* | 1.38 | *.143* | 0.69 | *.064* | 1.60 | *.039* |
| **Finnish citizenship** | 0.60 | *.153* | 0.92 | *.794* | 0.54 | *.081* | 0.72 | *.114* | 0.58 | *.008* | 0.44 | *<.001* |
| **Income support** | 1.11 | *.746* | 1.33 | *.270* | 1.18 | *.559* | 0.70 | *.064* | 1.22 | *.293* | 1.12 | *.553* |
| **Online respondent** | 0.14 | *.062* | 0.44 | *.212* | 0.46 | *.393* | 0.60 | *.083* | 0.47 | *.016* | 0.56 | *.076* |
| **Intercept** | 0.00 | *<.001* | 0.00 | *<.001* | 0.00 | *<.001* | 0.00 | *<.001* | 0.38 | *.244* | 0.15 | *.020* |

^a^OR, odds ratio.
