# Supplementary material for: Digital Information Technology Use, Self-Rated Health, and Depression: Population-Based Analysis of a Survey Study on Older Migrants
Source: J Med Internet Res. 2021 Jun 14;23(6):e20988. doi: 10.2196/20988 (PMC8240805; doi:10.2196/20988)
Supplement: Multimedia Appendix 3 [file jmir_v23i6e20988_app3.docx]

| **Appendix 3. Coeffiecients for depression and self-rated health (SRH). Fully adjusted models without weights.** | | | |
| --- | --- | --- | --- |
| **Daily internet non-use** | OR^a^ | | *P* |
| Depression | 2.97 | | *<.001* |
|  |  |  |  |
| **Smartphone non-use** | OR | | *P* |
| Depression | 2.11 | | *.003* |
|  |  |  |  |
| **Messages and calls non-use** | OR | | *P* |
| Depression | 2.03 | | *.009* |
|  |  |  |  |
| **Social media non-use** | OR | | *P* |
| Depression | 1.99 | | *<.001* |
|  |  |  |  |
| **Personal health data non-use** | OR | | *P* |
| Depression | 0.79 | | *.226* |
|  |  |  |  |
| **Health info non-use** | OR | | *P* |
| Depression | 0.92 | | *.674* |
|  |  |  |  |
| **Daily internet non-use** | OR | | *P* |
| Fairly good SRH | 2.43 | | .174 |
| Average | 2.66 | | .117 |
| Fairly poor or poor | 3.88 | | .046 |
|  |  |  |  |
| **Smartphone non-use** | OR | | *P* |
| Fairly good SRH | 1.94 | | .172 |
| Average SRH | 1.89 | | .168 |
| Fairly poor or poor SRH | 5.08 | | .002 |
|  |  |  |  |
| **Messages and calls non-use** | OR | | *P* |
| Fairly good SRH | 1.76 | | .256 |
| Average SRH | 1.44 | | .443 |
| Fairly poor or poor SRH | 3.50 | | .020 |
|  |  |  |  |
| **Social media non-use** | OR | | *P* |
| Fairly good SRH | 1.64 | | .057 |
| Average SRH | 1.36 | | .212 |
| Fairly poor or poor SRH | 2.00 | | .038 |
|  |  |  |  |
| **Personal health data non-use** | OR | | *P* |
| Fairly good SRH | 0.89 | | .651 |
| Average SRH | 0.63 | | .047 |
| Fairly poor or poor SRH | 0.65 | | .189 |
|  |  |  |  |
| **Health info non-use** | OR | | *P* |
| Fairly good SRH | 0.71 | | .169 |
| Average SRH | 0.71 | | .145 |
| Fairly poor or poor SRH | 0.73 | | .333 |

^a^OR, odds ratio.
